# Reward responsiveness in autism and autistic traits – Evidence from neuronal, autonomic, and behavioural levels

**DOI:** 10.1016/j.nicl.2023.103442

**Published:** 2023-05-24

**Authors:** Magdalena Matyjek, Mareike Bayer, Isabel Dziobek

**Affiliations:** a Berlin School of Mind and Brain, Humboldt-Universität zu Berlin, Luisenstr. 56, 10117 Berlin, Germany; b Institute of Psychology, Humboldt-Universität zu Berlin, Rudower Chaussee, 12489 Berlin, Germany

**Keywords:** EEG/ERP, Social cognition, Reward, Autism, ERP, Pupillometry

## Abstract

Autism has been linked to atypicalities in reward processing, especially in the social domain. However, results are heterogeneous, and their interpretation is hindered by the use of personally non-relevant social rewards. In this study we investigated behavioural (reaction times), neuronal (event-related potentials), and autonomic (pupil sizes) responses to personally relevant social rewards, money, and neutral outcomes in 26 autistic and 53 non-autistic subjects varying in levels of autistic traits. As hypothesised and preregistered, autism and autistic traits did not differently influence responses to social, monetary, or neutral outcomes on either response level. While groups did not differ in behaviour (reaction times), autism was linked to generally enhanced brain responses in early anticipation and larger pupil constrictions in reward reception. Together, these results suggest that when using personally relevant stimuli, autism is linked to generally preserved, although less neuronally efficient processing of rewards. Considering the role of social relevance in reward processing, we propose an interpretation of contradictory evidence from clinical practice and empirical research.

## Highlights

• Clinical practice and research yield contradictory evidence of the reward function in autism.
• Behavioural and autonomic data offer a broader context for the interpretation of brain data.
• Autism is linked to enhanced neuronal and autonomic processing but unaffected behaviour.
• Reward processing in autism is preserved, although less neuronally efficient.

## 1 Introduction

Rewards constitute a crucial factor in learning (Schultz, 2015) and thus are immensely important for life. The social motivation theory suggests that responsiveness to rewards in autism spectrum conditions (ASC) is atypical at least in the social domain (Chevallier et al., 2012). The consequence of this could be a cascade of neurodevelopmental difficulties including reduced pleasure from interacting with others, withdrawal from social situations, insufficient exposure to social stimuli, and finally social interaction difficulties. Thus, it was suggested that atypicalities in reward responsiveness may lie at the root of social difficulties in autism (Dawson et al., 2002, Dawson et al., 2005b).

In this framework, hypo-responsiveness (Chevallier et al., 2012) (quantified as hypoactivation of the reward circuit (Baumeister et al., 2023), diminished electrical brain activity (Kohls et al., 2011), decreased autonomic responses (Sepeta et al., 2012), or slower reactions (Demurie et al., 2011)) is speculated to be the underlying cause for the well-documented decreased behavioural motivation for social stimuli in autism. The first formulations of the social motivation theory predicted that reward responsiveness is diminished in autism especially in the social domain (Dawson et al., 2002, Dawson et al., 2005a, Schultz, 2005) but more recent works suggest general atypicalities in this group, manifesting in both social and non-social domains (Bottini, 2018, Clements et al., 2018, Keifer et al., 2021, Kohls et al., 2012).

However, empirical studies have yielded mixed results. While some report that autism is related to hypo-responsiveness specifically to social (Scott-Van Zeeland et al., 2010, Sepeta et al., 2012, Stavropoulos and Carver, 2014, Stavropoulos et al., 2014) or both social and non-social rewards (Baumeister et al., 2023, Kohls et al., 2011, Kohls et al., 2013, Kohls et al., 2014, Richey et al., 2014), other studies found no differences between ASC and comparison groups (Demurie et al., 2016, Ewing et al., 2013, Gilbertson et al., 2017). Moreover, some studies reported *hyper*-responsiveness to rewards in autism, especially to objects related to special interests (Cascio et al., 2014, Kohls et al., 2018, Watson et al., 2015), hinting at the importance of considering the role of personally relevant stimuli. Similarly, a recent *meta*-analysis of neuroimaging studies found both hypo- and hyper-activations in the reward brain circuit in the ASC group in comparison to non-autistic individuals (Clements et al., 2018). However, to support the prediction of *diminished* reward responsiveness in ASC, the results should show replicable and stable hypo-responsiveness to (social) rewards on multiple processing levels in autism. Thus, while any difference between the groups may be interpreted as *atypical* processing in autism, both enhanced and attenuated reward-related responses are not sufficient or clear evidence to support the claims of the social motivation theory. Moreover, several intervention programs for autism successfully use various social and non-social reinforces to promote desired behaviour (Kohls et al., 2012), which further contradicts the assumptions of aberrant reward processing in this group.

Although the social motivation theory has attracted considerable attention based on its attempt to explain autistic symptomatology, these heterogenous results cast doubts on its validity. To interpret these mixed findings, it is important to carefully consider the methodological aspects of published experimental designs (Bottini, 2018). Most crucially, familiarity and social relevance of the stimuli (e.g., faces) used as social rewards might be important but rarely addressed aspects in processing of social rewards. Often, studies use a smiling face of an unknown person as a social reward stimulus; However, such a face carries no relevance for the observer in the current study situation. In contrast to unknown faces, familiar and socially relevant faces are rated as more rewarding and elicit higher activation in reward-related brain structures (Acevedo et al., 2012, Bayer et al., 2021, Bayer et al., 2023, Matyjek et al., 2021, Sugiura, 2014). Moreover, familiarity of faces has the potential to improve otherwise atypical face processing in ASC (Pierce et al., 2004). Finally, numerous reports of autistic individuals and clinical observations contradict the assumed lack of social interest in autism (especially for relevant social partners, like friends, study or work peers, romantic partners; Jaswal & Akhtar, 2018). For these reasons, more research on the impact of socially relevant stimuli is needed. Here, we used pictures of the smiling face of the main experimenter, who is a familiar and relevant person in the study context (also see Hayward et al., 2018, Matyjek et al., 2020, Matyjek et al., 2021).

Additionally, when feedback in an experiment is delivered with a picture of a face, social anxiety traits in the participants should be controlled for, as anxious individuals are especially sensitive to social evaluation (Spain et al., 2018), which may confound the results. Moreover, social anxiety often co-occurs with autism (Spain et al., 2018) and has been previously linked to atypical reward processing (Richey et al., 2014). Therefore, here we controlled for the modulatory effects of social anxiety traits on reward responsiveness.

Further, even though autistic traits in the general population have been repeatedly related to atypicalities in reward processing (Carter Leno et al., 2016, Cox et al., 2015, Matyjek et al., 2020, Matyjek et al., 2021), studies rarely control for autistic traits in comparison groups. Importantly, autistic traits are distributed normally across the general population (Baron-Cohen et al., 2001, Hoekstra et al., 2007), they are aetiologically linked to autistic traits in ASC, and they seem to assess the same latent constructs in autism and non-clinical samples. Thus, studying effects of autistic traits on reward responsiveness in subclinical populations could assist in identifying relevant phenomena for ASC. Furthermore, neglecting autistic traits in the comparison groups may render it difficult to compare group effects between studies (and thus contribute to the inconsistencies in the literature). Therefore, to provide a broader picture of reward processing in the autism spectrum, in the current study we investigated it using both *population-based* and *psychopathological* approaches, i.e., we compared individuals with low levels of autistic traits (and no autism diagnosis) to those with high levels of the traits (and no diagnosis), and to individuals diagnosed with autism.

Finally, behavioural manifestations of social difficulties are key to the understanding of reward processing in autism. Yet, studies focusing on neuronal responses often lack a link between brain measures and behavioural manifestations in ASC (Baumeister et al., 2023, Kohls et al., 2013, Scott-Van Zeeland et al., 2010). Capturing indexes of reward responsiveness on multiple levels, however, has the potential to inform the interpretation of conflicting results in the literature and provides a more complete picture of the process. For example, behavioural indexes might aid the interpretation of co-recorded neuronal activity: Mixed results in studies investigating fusiform activation in response to observing faces in ASC may be explained by task demands, as shown by a negative correlation between the activation in this area and behavioural performance (Shafritz et al., 2015). Similarly, neuronal and autonomic measures have been shown to contribute differently to decision making and interoceptive attention (Al et al., 2020, Waschke et al., 2019). Although several indexes of reward responsiveness have been investigated in past research in the context of autism, including behavioural (e.g., reaction times, effort, accuracy; Demurie et al., 2011, Ewing et al., 2013, Geurts et al., 2008), neuronal (neuroimaging and electroencephalography (EEG); e.g., Kohls et al., 2011, Scott-Van Zeeland et al., 2010), and autonomic (e.g., electrodermal activity, pupil sizes; Neuhaus et al., 2015, Sepeta et al., 2012) levels, to our knowledge, no study to date has collected responses from all three levels from the same sample in a reward-related paradigm.

In the current study, we fill this gap by reporting behavioural indexes of reward responsiveness (combined measures of reaction times and accuracy), event-related potentials (ERPs), which offer excellent temporal resolution allowing for separate estimations of the two phases of reward processing, namely anticipation and reception (Berridge, 1996), and pupillary responses, which reflect the neuronal activation in the locus coeruleus (LC), a structure vastly involved in reward processing and motivation (Aston-Jones et al., 1999, Bouret and Richmond, 2015). In terms of targeted ERPs, reward reception was quantified by the P3, which is a positive potential peaking around 300 ms after stimulus onset at centro-parieto-occipital sites (more anterior for attention orienting and more posterior for stimulus evaluation and categorisation; Luck, 2005) and reflecting an elaborated cognitive and affective function linked to reward (Wu & Zhou, 2009). Based on our previous research, we divided reward anticipation into early and late phases (Matyjek et al., 2020, Matyjek et al., 2021). For those, respectively, we targeted the Contingent Negativity Variation (CNV) and the Stimulus Preceding Negativity (SPN). These ERPs are slow negative waves observed at various scalp regions depending on the task and peaking before a signal stimulus triggering a prompt action (CNV) or before receiving stimuli carrying important information, like feedback (SPN). Both CNV and SPN are linked to motivation and effort, and they reach higher amplitudes for anticipated affective or emotionally salient stimuli (Broyd et al., 2012, Brunia et al., 2012). As such, they have previously been used as indexes of reward anticipation (Matyjek et al., 2020, Matyjek et al., 2021, Stavropoulos and Carver, 2014, Stavropoulos et al., 2014). In terms of the autonomic responses, we target the pupil, as its size has been shown to vary with reward-related processes including attention allocation, effort, and anticipation of a reward (Carsten et al., 2021). The pupil has been observed to increase in size while anticipating rewards (Cash-Padgett et al., 2018, Koelewijn et al., 2018). In contrast, when receiving and evaluating outcomes, the pupil is negatively correlated with their reward values (Cash-Padgett et al., 2018, Matyjek et al., 2021). This pattern emphasises that these phases are not a unitary construct and have qualitatively different elements (Cash-Padgett et al., 2018). Although pupillometry is a promising tool for reward research, it has not yet been exploited it in the context of autism studies. For example, Sepeta et al., (2012) showed that typically developing, but not autistic children, showed pupil dilation to happy faces, but the children viewed the faces passively, without a reward context.

This study set out to investigate behavioural, neuronal, and autonomic responses in anticipation and reception of monetary and relevant social rewards as well as neutral outcomes across individuals with different levels of autistic traits and with autism without intellectual disability. In line with reward literature (Acevedo et al., 2012, Bayer et al., 2021, Matyjek et al., 2021, Sugiura, 2014), we assumed that familiar and relevant social stimuli are more motivating and have higher rewarding value than faces of unknown individuals often used in studies. This might normalise reward responses to social stimuli in autism (in line with the typical processing of familiar faces (Pierce et al., 2004) and preserved self-reported social interest in autism (Jaswal & Akhtar, 2018)), reducing the selectively social reward atypicalities predicted by the social motivation theory. Hence, in our modified design with a socially relevant context, we expected that on all processing levels (neuronal, autonomic, and behavioural), we would observe similar effects during anticipation and reception of social, monetary, and neutral incentives across groups and autistic traits (i.e., no interaction of group and reward type; Demurie et al., 2016, Ewing et al., 2013, Gilbertson et al., 2017, Neuhaus et al., 2015). Additionally, autism may be linked to employing generally enhanced neuronal resources in the formation of the stimulus-reward association in order to elicit behaviour similar to comparison groups (Groen et al., 2008, Matyjek et al., 2020, Matyjek et al., 2021, van Dongen et al., 2015). This would result in larger neuronal and autonomic responses in individuals with autism and high levels of autistic traits in contrast to those with low trait levels (i.e., a group effect in neuronal and autonomic, but not behavioural data). Further, the available research suggests that ASC-specific atypicalities in reward responsiveness are more pronounced in the anticipation (‘wanting’, stimulus-reward association, motivation) than reception ('liking’, experiencing pleasure) phase (Keifer et al., 2021, Kohls et al., 2012), as is the case in schizophrenia and addiction, placing atypical anticipation as a transdiagnostic marker of psychopathology (Kohls et al., 2012). Thus, we expected to see group differences in anticipation but not reception (Bottini, 2018). Specifically, based on our previous work discriminating early and late phases of reward anticipation (Matyjek et al., 2020, Matyjek et al., 2021), we expected that group differences (larger responses in the high autistic traits group/the ASC group) would be stronger in early than in late anticipation. Finally, in order to confirm that the targeted responses are reward-related, we predicted to see larger responses to rewarded conditions (social and monetary) in all measures, as compared to the neutral outcomes (Cox et al., 2015, Kohls et al., 2011).

In addition to testing these primary hypotheses, we aimed to explore several secondary analyses. First, to further quantify behavioural indexes of reward responsiveness, we collected scores estimating inhibition and approach tendencies from the participants and aimed to relate them to the neuronal and autonomic measures as well as autistic traits. Second, although we were primarily interested in *reward* responsiveness and for that the primary analyses were conducted on the data from successful trials (where reward could be obtained), we also explored the neuronal and autonomic responses in the reception of unsuccessful (non-rewarded) trials. Finally, for a dimensional analysis of autistic traits (instead of group-based), we explored whether the trait levels across all participants predict the reward-related reaction times, ERPs, and pupillary responses in linear and non-linear models.

## 2 Methods

The methods, hypotheses, and analyses were preregistered at https://osf.io/3re72. Data, analysis code in R, and an html file including all analyses' steps and results can be found at https://osf.io/vse38/.

### 2.1 Sample size determination

To estimate the sample size, we performed a power analysis with the g*power software (Faul et al., 2009), with power set to 0.8 and with an intermediate effect size f = 0.302. The effect size was calculated from the between-subject factor of group (high vs. low autistic traits) in response to reward cues in our previous experiment (Matyjek et al., 2020, Matyjek et al., 2021). This analysis yielded a total sample size of 52 with 26 data sets in each of two groups planned for comparisons (ASC vs. low autistic traits, and high vs. low autistic traits). Based on this, we planned to recruit 26 participants per group, summing up to the total of 78 participants.

### 2.2 Participants

A total of 82 volunteers across three groups (ASC, low- and high autistic traits) participated in the study. All participants were White Europeans speaking German. The data sets of 3 participants (two from the ASC group) were excluded due to poor EEG signal quality (1), refusal to perform a task involving money (1), and a technical issue with EEG recording (1). Demographic information for all groups with group comparisons are summarised in Table 1. All participants provided written informed consent. The study was approved by the ethics committee of the Faculty of Psychology of the Humboldt-Universität zu Berlin and was conducted in accordance with the Declaration of Helsinki. Participants were compensated 8 Euros per hour plus additional 4 Euros as a monetary reward earned during the task (for details, see section 2.3), which resulted in a total of approx. 30 Euros.

**Table 1:** Demographic and trait characteristics of subject samples in all groups. Count is provided for gender and means (with standard deviations) for all other items. HAQ = high autistic traits, LAQ = low autistic traits, ASC = Autism Spectrum Disorder, AQ = Autism Spectrum Quotient score, BIS/BAS = Behavioural Inhibition/Approach System, LSAS-SR = Liebowitz Social Anxiety Scale, R:N/L = Right, Neutral/Left (handedness), OR = odds ratio in Fisher's Exact Test. Statistically significant tests were marked with ** for p < 0.01 and *** for p < 0.001.

**Group description**			
	LAQ *N* = 26	HAQ *N* = 27	ASC *N* = 26
Gender – female:male	18:8	12:15	13:13
Age (years)	30.6 (7.9)	31.9 (10.6)	32.7 (10.7)
AQ (total)	10.9 (2.3)	25.9 (5.5)	39.3 (5)
BAS drive	12.5 (2.1)	11.6 (1.9)	11.2 (2)
BAS reward responsiv.	16.8 (1.7)	15.7 (2.3)	14.9 (2.3)
BAS fun seeking	12.5 (1.8)	11.2 (1.9)	9.7 (2.7)
BIS	18.8 (3.2)	20.9 (4.7)	23.5 (3.8)
LSAS-SR	31.1 (15.9)	58.6 (27.2)	83.5 (32.2)
Handedness – R:N/L	25:1	25:2	20:6

**Between-group differences**			
	LAQ vs. HAQ	LAQ vs. ASC	
Gender	*OR* = 2.76	*OR* = 2.21	
Age (years)	*t* = -0.51	*t* = -0.79	
AQ (total)	*t* = -13.02^***^	*t* = -26.25^***^	
BAS drive	*t* = 1.71	*t* = 2.35*	
BAS reward respons.	*t* = 1.87	*t* = 3.42^***^	
BAS fun seeking	*t* = 2.68*	*t* = 4.43^***^	
BIS	*t* = -1.93	*t* = -4.93^***^	
LSAS-SR	*t* = -4.51^***^	*t* = -7.42^***^	
Handedness	*OR* = 0.51	*OR* = 6.25	

#### 2.2.1 Non-autistic participants

Non-autistic participants were recruited via internet advertising platforms and flyers distributed at Berlin’s university campuses. Inclusion criteria were age (18–50), proficiency in German, no history of psychological, neurological, or psychiatric disorders in the last 6 months (including medication), and no past diagnosis of such. Interested volunteers were asked to complete the Autism Spectrum Questionnaire (AQ; Baron-Cohen et al., 2001) and were invited to participate based on the score (we aimed to increase the spread of the scores and to balance the size of low and high scoring groups). The mean AQ score in the non-autistic group was 18.6 (*SD* = 8.7); groups with high (HAQ) and low (LAQ) autistic traits were created based on a median split (*Mdn* = 17). This sample had a mean age of 31.3 (*SD* = 9.3). All participants had normal or corrected-to-normal vision and 50 were right-handed. One participant reported attending a psychotherapy in the last six months, and two earlier than that. No participants in this group had been medicated with psychopharmaceuticals.

#### 2.2.2 Autistic group

Participants with ASC were recruited via specialised autism outpatient clinics and hospitals in Berlin. All participants had a prior diagnosis matching the DSM-5 criteria for autism spectrum disorder (22 Asperger’s, one atypical autism, and three autism spectrum disorder) made by professionals in specialised autism-diagnosis centres (the diagnosis was confirmed directly by the centres and/or by a written diagnosis provided by the participants). All participants were verbal and did not have an intellectual disability. In 20 cases, data were available for the Autism Diagnostic Observation Schedule (ADOS; Lord et al., 2000), and in 17 also for the Autism Diagnostic Interview-Revised (ADI-R; Bölte & Poustka, 2001). Additionally, inclusion criteria were age (18–50) and proficiency in German. All participants had normal or corrected-to-normal vision and 20 participants were right-handed. Several participants in the ASC group reported co-occurring psychopathology and/or receiving psychotherapy in the last six months (N = 6), and earlier (N = 5) (all for depression and/or anxiety). Four participants were medicated at the time of the study or in the last six months and two more earlier than that (all with selective serotonin or serotonin-norepinephrine reuptake inhibitors).

### 2.3 Stimuli and task

**Fig. 1:**
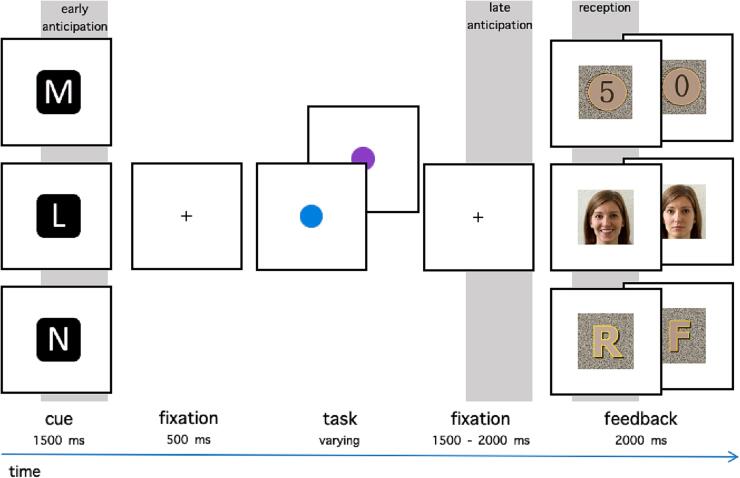
Trial structure. The possible reward in a trial was signalled with an incentive cue: M for monetary (German: Münze), L for social (German: Lächeln) and N for neutral (no reward; German: neutral). In the task, participants were asked to respond as fast as possible to a blue or a purple target with corresponding keys. In successful trials, feedback with 5-cent coin, a smiling face, or a letter “R” (correct; German: richtig), was presented in monetary, social, and neutral condition, respectively. Incorrect trials were indicated with 0-cent coin, neutral face, or a letter “F” (incorrect, German: falsch). The shaded rectangles mark approximate time windows for ERPs in early and late anticipation, and reward reception. (For interpretation of the references to colour in this figure legend, the reader is referred to the web version of this article.)

We adapted a cued incentive delay task (Knutson et al., 2000) to include both social and non-social rewards. Participants were shown a cue indicating a possible reward in each trial and were asked to speedily respond to a following target. Fig. 1 depicts the overall course of a trial. The instructions were delivered both in writing and verbally.

Each trial started with a cue presented at the centre of the screen for 1500 ms, which indicated the condition: social (S), monetary (M), or neutral (N). The cues consisted of informative white letters (see Fig. 1) on black squared background (4 × 4° of visual angle). The cue was followed by a white fixation cross (30 × 30 pixels) displayed centrally for 500 ms. Then, a blue or a purple target (a circle, 1 × 1° of visual angle) was displayed in the centre. The display time of the targets was adapting to each participant’s performance: it was increased by 20% if in the last four trials no more than 1 response was correct, kept the same if 2 responses were correct, or decreased by 20% when 3 or 4 responses were correct (resulting in approx. 60% accuracy). The colour of the target in each trial was random and the response keys corresponding to the colours were counterbalanced across participants. A trial was successful when participants pressed the correct button during the display of the target. After the button press (or the end of the display time), the pre-feedback waiting period with a fixation cross was presented for 1500 to 2000 ms (jittered across trials), followed by a feedback stimulus (matching the condition) presented for 2000 ms.

Correct responses were rewarded with a picture of a happy/approving face of the main experimenter in S, a picture of a “5″ coin in M, and a letter “R” (for German *richtig* meaning *correct*) in N. Incorrect responses in S, M, and N conditions were followed by a face with a neutral expression, a “0” coin, and letter “F” (for *falsch*, or *wrong*). Letters in N and coins in M were displayed on a background made of scrambled pixels of the S rewarding feedback picture (the happy/approving face). All feedback stimuli were equal in size (4 × 4° of visual angle) and luminance (ensured with the mean value of luminance in perceptual space in GIMP 2.0, which was additionally confirmed with a photometer). Participants were instructed that a “5” coin meant they were receiving additional 5 cents.

Each of the six blocks consisted of one condition. The blocks were presented pseudo-randomly: The first three blocks were presented in random order and the last three blocks repeated that order. Each block consisted of 50 trials, resulting in a total of 300 trials. Before the start of the experiment, 30 trials across conditions were presented as training.

### 2.4 Procedure and socialising

After signing the consent form, the participants were prepared for the EEG recording (approx. 20 min). During this time the experimenter had a light social conversation following a semi-scripted interaction. The aim was to provide a natural acquaintance with the experimenter, with whom all participants spent the same amount of time. Moreover, this allowed the participants to familiarise with the experimenter’s face in a natural fashion: from various angles and with various facial expressions. To emphasize the shared social context, the experimenter also indicated that this research was her project, and that she appreciated the subjects’ participation in the study. Then, participants were seated in an electrically shielded room at 70 cm from a 19-inch computer screen and 85 Hz refresh rate. To keep the light conditions constant, the room was artificially lit. Participants were asked to place their chin and forehead on a headrest to restrain movements. The experiment was programmed and executed in MATLAB. Participants were asked to identify the person on the pictures used in the social condition prior to the training and all correctly recognised the experimenter. After the recording, participants answered several debriefing questions on a computer screen. At the end they were debriefed and informed about in details about the purpose of the study.

### 2.5 Behavioural measurements

#### 2.5.1 Reaction times

Both shorter reaction times and higher accuracy have been previously interpreted as indexes of increased motivational and reinforcing values of the related rewards (Neuhaus et al., 2015). Since faster responses may lead to lower accuracy, increasing response time to ensure more successful trials (and thus rewards) may be used as a strategy. Therefore, we targeted reaction times corrected for accuracy, i.e., the linear integrated speed-accuracy score (LISAS; Vandierendonck, 2018).

#### 2.5.2 Debriefing questions

We collected self-reported measures of general motivation, importance of reward type, sense of agency, and the motivational value of cues as well as rewarding value of feedback stimuli. See supplementary material for details.

#### 2.5.3 Questionnaires

To quantify autistic traits, we used the Autism Spectrum Quotient (AQ; Baron-Cohen et al., 2001) and the Social Responsiveness Scale (SRS; Constantino & Gruber, 2005), which were significantly correlated in our sample, *r*(65) = 0.66, *p* < 0.001. However, because 14 participants did not feel comfortable with the SRS (in contrast to the self-reported AQ, the SRS is completed by another person), we did not focus on these data any further.

To quantify further reward-related behaviour, we collected the Behavioural Inhibition and Approach Systems Scale (BIS/BAS; Carver & White, 1994), and to control for social anxiety traits in all statistical models, we used the Liebowitz Social Anxiety Scale self-reported (LSAS-SR; Liebowitz, 1987). The mean scores for each group are presented in Table 1.

### 2.6 EEG data acquisition and pre-processing

The continuous EEG signal was recorded from 64 silver/silver-chloride active scalp electrodes (Biosemi Active Two) at a sampling rate of 512 Hz. An elastic cap with the extended 10–20 international electrode placement system was used. The collected signals were referenced online to the CMS-DRL ground loop. The electrode offsets were kept within the range of ± 20 µV. Six external electrodes were used: Four electrodes were placed at the outer canthi and below the eyes (to collect the horizontal and vertical electro-oculograms) and two were placed on the mastoids. Data was filtered online with a 100 Hz low-pass and 0.01 Hz high-pass filter.

The offline pre-processing steps were performed using BrainVision Analyzer (Brain Products GmbH, Munich, Germany), in which all signals were re-referenced to average reference and filtered with a low-pass filter of 40 Hz (slope 8 dB/oct). The continuous data were segmented into segments ranging from −100 ms before, to 7500 ms after the cue onset. A pre-cue baseline of 100 ms was applied. To identify and remove blinks and eye movements, an independent component analysis algorithm (restricted fast ICA) was used. Channels with low quality and noisy signals were interpolated using spherical splines of order 4 (2.7 % of all channels). The data were divided into three sub-segments representing the phases: early anticipation (-100 ms before to 1500 ms after the cue onset), late anticipation (–600 ms before feedback onset to the feedback onset), and reception (- 100 ms to 2000 ms after the feedback onset). Further, to exclude artifacts, a semi-automatic procedure was applied targeting signals exceeding ± 100 µV or voltage steps larger than 100 µV, which led to rejection of 5–6 % of signals across phases. The number of artifact-free segments did not vary significantly between conditions in either of the phases; see Table S1 in supplementary material for details).

The temporal windows and regions of interest for the brain responses were chosen based on prior research and visual inspection of grand averages. For the early and late anticipation phases the time windows were respectively the CNV and the SPN defined as the last 500 ms of each phase: in early anticipation this was 1000 – 1500 ms after cue onset (with −100 – 0 ms baseline), and in the jittered late anticipation phase this was −500 – 0 ms time-locked to feedback onset (with –100 – 0 ms baseline locked to the onset of the pre-feedback fixation cross). In the reception, the P3 was identified from 230 to 500 ms after feedback onset. Mean amplitudes for these time windows were calculated from electrodes Pz, P1, P2, POz, PO3, PO4, Oz, O1, O2. Finally, the mean amplitudes of the CNV, SPN, and P3 were aggregated per participant and condition and used in the statistical analyses.

### 2.7 Pupillary data acquisition and pre-processing

Pupillary responses were recorded binocularly with a desktop-mounted eye tracker (Eye Tribe, TheEyeTribe) with a 60 Hz sampling rate. The EyeTribe Toolbox for Matlab was used to send event triggers. The calibration was conducted with a nine-point grid and accepted when accuracy of < 0.7 degree was achieved. Data sets with poor data quality (i.e., more than 50% missing trials, with a trial removed when missing over 50% samples) were excluded from further processing and analysis (13 sets). Offline preprocessing was performed with Matlab code published by Kret & Sjak-Shie (2018) with their default settings (but upsampling was reduced from 1000 to 100 Hz). This includes blink and missing data interpolation, filtering and smoothing. In the remaining 66 datasets (in LAQ, HAQ, and ASC: 23, 24, and 19, respectively), the number of clean trials did not differ significantly for any two conditions across phases (see Table S1 in supplementary material for details). Then, using a custom code in R ver. 4.0.2 (R Core Team, 2020), segmentation of the signal into phases with a subtractive baseline correction was performed: −200 to 0 ms before the cue onset for early anticipation, and −200 to 0 ms before the pre-feedback for late anticipation and for reception. Finally, the mean pupil size was calculated for each segment: 0 to 1500 ms after cue onset (early anticipation), −1500 to 0 ms before feedback onset (late anticipation), and 0 to 2000 ms after feedback onset (reception) and aggregated for participants and conditions.

### 2.8 Data analysis

All data analyses were performed using R ver. 4.0.2 (R Core Team, 2020). The significance level for all the tests was set to 0.05.

#### 2.8.1 Primary analyses

As registered, we analysed reward-related ERPs, pupil sizes, and reaction times corrected for accuracy in two approaches: 1) *population-based approach*, which includes individuals with high levels of autistic traits as compared to individuals with low levels of autistic traits; and 2) *psychopathological approach*, which includes individuals diagnosed with autism as compared to individuals with low trait levels. Participants’ responses to the debriefing questions were analysed across the three groups, with Pearson’s correlation or linear models.

For reaction times, brain, and pupillary responses, we built multiple regression models with mixed effects (random intercepts for subjects) with the lmerTest package ver. 3.1–2 (Kuznetsova et al., 2017). Models which violated regression assumptions were considered for outliers (based on influence and deletion diagnostics) and re-fitted after either overwriting a data point with the group’s mean in the given condition, or exclusion of a subject’s data set. For the estimation of the main effect of condition, an analysis of variance (ANOVA) with Satterthwaite approximation for degrees of freedom was calculated on the models. For all reported post-hoc tests, a Holm correction was applied. Partial Cohen’s *f* (*f_p_*) from ANOVAs performed on the models with the effectsize package ver. 0.3.2 (Ben-Shachar et al., 2020) were calculated as approximate effect sizes. As registered, we controlled for social anxiety traits by including the (centred) LSAS-SR score in all the models. Because our hypothesis was that groups would respond similarly to different conditions (i.e., no interaction of group and condition), we first checked whether the interaction term was statistically significant. Since an insignificant effect does not mean a true negative effect, we used Bayes factors (BF) to provide an explicit quantification of evidence in favour of a model without the interaction vis-à-vis a model with the interaction (Kass & Raftery, 1995). In case of strong evidence in favour of a model without the interaction term, we continued the analysis with the model including only main effects.

#### 2.8.2 Secondary analyses

Methods and results for all secondary analyses are presented in the supplementary material. As registered, we explored (1) correlations between questionnaires (AQ, BIS/BAS, and LSAS-SR), ERPs, and pupil sizes, and (2) reaction times, ERPs, and pupillary responses also in unsuccessful (not rewarded) trials. Additionally to the registered analyses, we performed (3) dimensional analyses of AQ as a predictor of reward-related responses across all participants, and (4) exploratory analyses of the effects of age and gender in all the primary models.

## 3 Results

### 3.1 Primary analyses

**Fig. 2:**
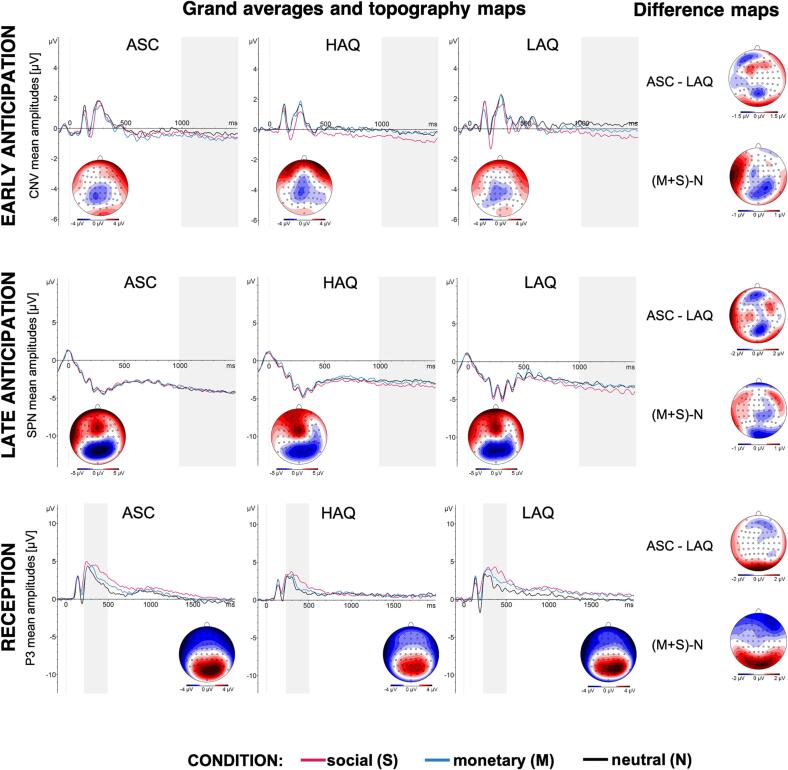
Grand averages of ERPs. The panels show the grand averages for groups (low autistic traits, LAQ; high autistic traits, HAQ; and autism, ASC) and conditions (social, S; monetary, M; and neutral, N) for each reward processing phase: early anticipation (top panel), late anticipation (middle panel), and reception (bottom panel). All panels show the mean activity in the region of interest (electrodes Pz, P1, P2, POz, PO3, PO4, Oz, O1, O2). The dotted vertical lines mark the onset of each phase: cue presentation in early anticipation, fixation cross in late anticipation, and feedback in reception. The grey rectangles mark the time windows for analyses. Note that for the purpose of visualisation, the SPN in the late reception is plotted as aligned to the onset of the fixation cross until 1500 ms, even though the display time was jittered (1500 – 2000 ms) and the analysis included the last 500 ms before the feedback onset in each trial.

**Fig. 3:**
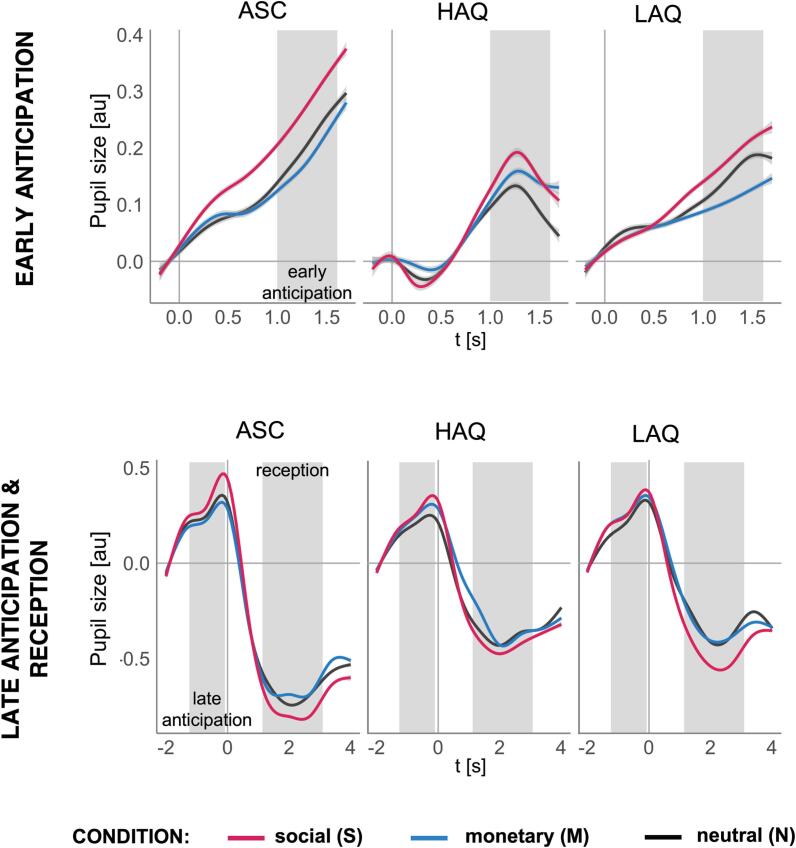
Grand averages of pupillary responses. The panels show the grand averages for groups (low autistic traits, LAQ; high autistic traits, HAQ; and autism, ASC) and conditions (social, monetary, and neutral) for each reward processing phase: early anticipation in the top panel, and late anticipation with reception in the bottom panel. The plots were created with generalized additive model smoothing and the grey shades show 95% confidence interval of this fit. The grey shaded areas mark time windows used for analyses. For visualisation purposes in the reception, the following 1000 ms of the intertrial interval were plotted. In the joint plot for late anticipation and reception 0 marks the onset of the feedback.

The grand averages of the brain and pupil responses are shown in Fig. 2 and Fig. 3, and mean responses across conditions, groups, and phases are shown in Table S2 in the supplementary material.

As predicted, in none of the models we observed an interaction effect of group (LAQ, HAQ, ASC) and condition (S, M, N), all *F* <= 2.09, all *p* >= 0.13. In all analysis we found strong evidence in favour of models without the interaction term (all *BF* >= 20) (Kass & Raftery, 1995) and those models showed a better fit (based on BIC) than models including this term. Hence, in the following we report only results of models re-fitted without the interaction term (nevertheless, all analyses steps can be found in the code).

#### 3.1.1 Population approach (low AQ vs. High AQ)

##### 3.1.1.1 Reaction times

The factor condition significantly predicted reaction times corrected for accuracy, i.e., LISAS scores, *F*(2,100) = 11.7, *p* < 0.001, *f_p_* = 0.48, with fasted responses in M than N (*p_corr_* < 0.001, *est* = 9.99) and M than S (*p_corr_* = 0.001, *est* = 7.37). Group was not a significant predictor (*f_p_* = 0.13). For details and additional analyses of uncorrected reaction times and accuracy, see the code/html file.

##### 3.1.1.2 ERPs

Analyses of the CNV in the early anticipation, the SPN in the late anticipation, and the P3 in the reception all yielded a main effect of condition, all *F*(2,106) >= 4.61, *p* <= 0.012, *f_p_* >= 0.3, with larger ERP responses (i.e., more negative CNV and SPN, and more positive P3) for S than N (all *p_corr_* <= 0.012, all *est* >= 0.61), and, in SPN and P3, for S than M (all *p_corr_* <= 0.04, all *est* >= 0.49). Additionally, in the reception, P3 was also statistically significantly larger for M than N (*p_corr_* = 0.002, *est* = 0.59). Group was not a statistically significant predictor in these models (all *f_p_* <= 0.18).

##### 3.1.1.3 Pupil sizes

Condition was a statistically significant predictor of pupil sizes in early anticipation, *F*(2,90) = 5.74, *p* = 0.004, *f_p_* = 0.36, and in late anticipation, *F*(2,92) = 3.88, *p* = 0.024, *f_p_* = 0.29 (in reception *f_p_* = 0.19). In both anticipation phases pairwise post-hoc tests revealed that this effect was driven by larger dilations to S than N (all *p_corr_* <= 0.035, *est* >= 0.04) and in early anticipation also to S than M (*p_corr_* = 0.003, *est* = 0.05). Group did not significantly predict pupil size in any model (all *f_p_* <= 0.1).

#### 3.1.2 Psychopathological approach (low AQ vs. ASC)

##### 3.1.2.1 Reaction times

The analysis of reaction times yielded a significant effect of condition, *F*(2,88) = 3.64, *p* = 0.03, *f_p_* = 0.29, with faster responses in M than in S (*p_corr_* = 0.037, *est* = 5.74). Group did not predict the responses, *f_p_* = 0.04. For details, see the code/html file.

##### 3.1.2.2 ERPs

The models yielded a main effect of condition in early anticipation, *F*(2,104) = 5.25, *p* = 0.007, *f*_p_ = 0.32, with larger CNV to S (*p_corr_* = 0.006, *est* = 0.45) and M (*p_corr_* = 0.03, *est* = 0.36) in comparison to N, and in reception, *F*(2,104) = 20.79, *p* < 0.001, *f_p_* = 0.63, with the largest P3 amplitudes to S, than to M, and smallest to N (all *p_corr_* <= 0.004, all *est* >= 0.64). Condition did not predict the SPN amplitudes in late anticipation (*f_p_* = 0.17). Group was a significant predictor only in early anticipation, *F*(1,52) = 4.83, *p* = 0.032, *f_p_* = 0.31, where the CNV amplitudes were larger in the ASC than in the LAQ group (effect sizes for group in late anticipation and in reception were *f_p_* <= 0.16).

##### 3.1.2.3 Pupil sizes

Condition predicted pupil sizes in early anticipation, *F*(2,84) = 5.57, *p* = 0.005, *f_p_* = 0.36, and in late anticipation, *F*(2,82) = 5.63, *p* = 0.004, *f_p_* = 0.37 (in reception *f_p_* = 0.21). Pupil sizes were larger to S than N in both anticipation phases (all *p_corr_* <= 0.033, *est* >= 0.04) and to S than M in early anticipation (*p_corr_* <= 0.03, *est* >= 0.05). Group significantly predicted pupil sizes only in reception, *F*(1,42) = 7.27, *p* = 0.01, *f_p_* = 0.42, with larger constrictions in ASC than LAQ (in anticipation phases both *f_p_* <= 0.17).

#### 3.1.3 Debriefing questions

Groups did not differ in general motivation or sense of agency in the task, but reward type was more important to participants with higher autistic traits. Ratings of the motivational values of the cues and the rewarding values of the feedback stimuli revealed that while all groups found stimuli in S and M more motivating and rewarding that in N, the ASC group’s ratings were lower than in the other groups. See supplementary material for details.

### 3.2 Secondary analyses

To summarise the highlights of the secondary analyses (see the supplementary material), we found that autistic traits were positively correlated with social anxiety traits and with stronger behavioural motivation to move away from unpleasant stimuli than to move towards desired outcomes (as assessed with the BIS/BAS). Further, the dimensional analyses (with AQ instead of group) paralleled the primary analyses: AQ did not interact with condition in either measure or phase, but higher AQ scores were linked to enhanced neuronal and pupillary responses respectively in early anticipation and in reception. Additional analyses revealed that the best fit for the relationship between AQ and all measures is linear, which suggests that autistic traits play a similar role in reward processing across individuals with and without autism. Finally, enhanced brain and pupillary responses were correlated across processing phases (early and late anticipation, reception), but not with each other.

## 4 Discussion

In this study, we investigated responsiveness to relevant social rewards, money, and neutral outcomes across autistic traits and in individuals with autism spectrum diagnosis. By addressing multiple levels of reward processing – neuronal, autonomic, and behavioural – we aimed to grasp a bigger picture of the process, which is necessary for an informed interpretation of reward atypicalities in ASC. The most important finding in this study is that autism was linked to enhanced neuronal (early anticipation; larger CNV) and pupillary (reception; larger constrictions) processing of rewards, but typical performance (reaction times). While these results contradict accounts suggesting *reduced* responsiveness in ASC to social and non-social rewards (Bottini, 2018, Clements et al., 2018, Keifer et al., 2021, Kohls et al., 2012), they are not isolated in the literature (Dichter et al., 2012, Groen et al., 2008, Matyjek et al., 2020, Matyjek et al., 2021, Pankert et al., 2014, van Dongen et al., 2015). To interpret them, functional meaning of neuronal activation in the light of behaviour needs to be considered.

Just as there are two possible interpretations of *diminished* reward responsiveness on the neuronal level: functional deficit (Scott-Van Zeeland et al., 2010) or higher efficiency in neuronal processing (Shafritz et al., 2015), *enhanced* responses may be attributed to either functional superiority (versus the comparison group) or less efficient neuronal processing. Because there were no behavioural differences between groups in this study, it is presumable that the enhanced early processing in autism reflects the rapid formation of reward representations and the initial anticipatory processes. Similarly, increased autonomic measures in reward reception indicate preserved processing of the feedback (cf. with results from (Baumeister et al., 2023), who observed hyperactivation of the ventral striatum during reward reception in over 200 ASC participants, although only at an uncorrected level). Hence, the interpretation of functional (e.g., translatable to behaviour) group differences is not likely. Instead, it is more reasonable that neuronal hyperresponsiveness to rewards in autism reflects less efficient neuronal processing in the sense that larger brain activation is required to achieve similar performance (Shafritz et al., 2015). In that sense, the translation from biology to behaviour seems atypical in autism, demanding heightened neuronal and autonomic responses to attain comparable behavioural responses.

Importantly, the enhanced neuronal and autonomic processing in our data was predicted by levels of autistic traits across all participants, which quantify manifestations of socio-communicative, attentional, and imagination-related behaviours characteristic for ASC (Baron-Cohen et al., 2001). This suggests that the enhanced reward responsiveness on the neuronal and autonomic levels is linked to more pronounced autistic behavioural expressions, which cuts through the borders of diagnostic groups. This speaks for the value of the dimensional analysis of autistic traits in addition to the coarse group differences based on the diagnostic cut-off. In this vein, by exploring autistic traits as continuously distributed in the population, we showed that reward processing atypicalities are likely linked to these traits in a linear manner: the higher the autistic traits, the larger the reward-related responses. Together, these results suggest that the neuronal hyperresponsiveness in autism and higher autistic traits is a marker of existing, albeit less neuronally efficient, reward processing.

Although the social motivation theory proposes that autism is characterised by diminished reward processing (i.e., hypo-responsiveness), likely specifically in the social domain (Dawson et al., 2002, Dawson et al., 2005a, Schultz, 2005), we found that behavioural, neuronal, and autonomic responses were not differently influenced by the type of outcome across the groups (i.e., no interaction of group and reward type). Moreover, by using Bayes factors for the models of all reward responses (neuronal, pupillary, and reaction times), we found strong evidence in favour of no interaction being the true effect in all the data.

A critical element in our design that should be considered is the relevance of the social rewards. While a common social stimulus in reward paradigms is a picture of a smiling, unknown person (Kohls et al., 2013, Scott-Van Zeeland et al., 2010, Stavropoulos and Carver, 2014), in this study we used photographs of the main experimenter. The experimenter’s face became *familiar* to the participants during the study preparations, which they confirmed by recognising her in the photographs prior to the task. Importantly, the experimenter was also *socially relevant* in the context of the study, as she provided explanations and instructions, engaged in a semi-scripted, casual social exchange, and was present in the laboratory throughout the course of the study. Therefore, while many previous studies used faces that were irrelevant in the study situation (even familiar faces, but absent or irrelevant in the context of the task; (Neuhaus et al., 2015, Pankert et al., 2014, Stavropoulos et al., 2014)), we created a relevant social context. There is accumulating evidence suggesting that faces which are more familiar and relevant elicit higher activation in the brain reward structures (Acevedo et al., 2012, Bayer et al., 2021). Moreover, familiar, but not unfamiliar faces, have been reported to elicit neuronal and pupillary responses in ASC similar to those of comparison groups (Nuske et al., 2014, Pierce et al., 2004). Hence, we propose that the relevance of the social stimuli used in this study is an important qualitative factor which could have improved otherwise atypical responsiveness (as observed in other studies using unfamiliar faces; Kohls et al., 2011, Scott-Van Zeeland et al., 2010, Stavropoulos and Carver, 2014) to social rewards in ASC and higher autistic traits.

Together with the enhanced neuronal processing of rewards in autism, the context of social relevance in this study provides a coherent interpretation of our results in contrast to the social motivation theory: Autism is characterised by preserved, albeit less neuronally efficient, motivation and liking of non-social and relevant social (i.e., a smile of a relevant interaction partner) incentives. This relevance may be a critical element, explaining why in other studies autistic individuals show diminished brain responses in contrast to comparison groups while processing faces of unknown and absent people: They are simply not important enough in the context to engage the costly neuronal resources. Thus, the relevance of social rewards offers a bridge between the contradictory assumptions of the social motivation theory and the evidence coming from successful reward-based intervention programs (Kohls et al., 2012). Future studies should test this interpretation by systematically varying social relevance of rewards.

Further, the current study design, although based on a well-established paradigm (cued incentive delay task; Knutson et al., 2000) includes several aspects which allow us to disentangle potentially confounding factors in reward processing and can thus contribute to the understanding of the mixed results in the literature. Firstly, we used symbolic incentive cues which were not themselves rewarding (in contrast to showing a coin or a smiling face as a cue; Kohls et al., 2011). Thus, we ensured that the responses in early and late anticipation were indeed reflecting reward anticipation and not reception. Further, we included a non-rewarded condition (neutral), in which informative feedback was provided, but which did not offer any external rewards. Due to this, the observed enhanced responses to the social and non-social conditions in contrast to the neutral outcomes can be interpreted as reward processing on top of feedback processing. Finally, in all statistical models we controlled for social anxiety traits, as it is linked to atypical reward processing (Richey et al., 2014), and correlates with autistic traits (see point 1.1. in the supplementary material). This allowed us to interpret the obtained results as more autism-specific.

While more research is needed before firm conclusions can be drawn, our data suggest that the neuronal, autonomic, and behavioural indexes of reward processing reflect distinct mechanisms and together offer a broader picture of this function. In line with this, ERPs and pupillary responses across conditions did not correlate with each other, but in each processing level (neuronal and autonomic), we observed consistent correlations between reward phases. The SPN was positively associated with the CNV (both negative ERPs) and negatively with the P3 values, which suggests that the larger the late anticipation, the larger both the early anticipation and the reception of rewards. The pupil sizes in the reception phase correlated negatively with the pupil sizes in early anticipation, which suggests that the larger the anticipation (indexed as increased dilations), the larger the reception (indexed as increased constrictions). These consistencies emphasise the additive explanatory values of ERPs and pupil sizes and emphasise the importance to investigate reward function on multiple levels.

At the same time, several limitations in this study should be noted. First, we focused on adults, but reward processing atypicalities linked to autism have been shown primarily in childhood (Kohls et al., 2011, Scott-Van Zeeland et al., 2010, Stavropoulos and Carver, 2014) and are possibly dynamic throughout development (Keifer et al., 2021). In additional explorative analyses we observed that age was linked to diminished ERP responses in late anticipation and reception (see Figure S7 in the supplementary material). Similarly, exploratory models yielded that females exhibited increased pupil responses in late anticipation (dilations) in comparison to males (see Figure S8 in the supplementary material). However, it should be noted that groups in this study did not differ in age or gender distribution. Nevertheless, here we used only one face (White, female) and future studies should consider using more diverse social stimuli to systematically explore gender effects. Furthermore, for the dimensional analyses we used the full AQ scores, even though the social subscale of the AQ or the SRS would provide a more direct test of the social motivation theory’s predictions. This was not possible in our data, because many participants with ASC were not comfortable with the SRS, which needs to be filled out by a close person, and several provided a pre-existing full AQ score (from which we could not calculate the subscales’ scores). It should also be noted that compared to non-autistic peers, autistic individuals show atypicalities in face processing, e.g., lower recognition and emotion discrimination, altered gaze patters to face regions, and different cortical specialisation to faces (Dawson et al., 2005a). Nevertheless, none of these aspects are likely to be confounding the results in our study: All participants correctly recognised the main experimenter and her emotional expression in the social stimuli prior to the task; The stimuli were limited in size to mitigate the potential differences in focusing the gaze on mouth or other face regions, and we observed no neuronal differences in the processing of social stimuli between the groups. Most importantly, as suggested by previous research (Pierce et al., 2004), it is possible that by using socially relevant stimuli we limited the face processing differences between the groups and were able to instead capture reward processing differences. Finally, due to the need to maintain high experimental control over luminance and onset timing of the stimuli (for pupillometry and ERPs), we used static stimuli, even though these stimuli are characterised by reduced ecological validity, especially in the social domain (Dziobek, 2012). Thus, future studies should attempt to replicate our results with dynamic stimuli.

Another interesting point concerns the seeming inconsistency across our different measures, with similar (ERPs) or enhanced (pupils) responses to feedback in the ASC group and, on the other hand, decreased ratings of the stimuli’s reward value in the autistic group. We tentatively suggest that the autistic and comparison groups might have interpreted the extremes of the rating scale differently: While non-autistic participants might have interpreted “very rewarding” as ‘most rewarding in the study context’, autistic individuals might have used a more general interpretation, thus resulting in lower rating values. Nevertheless, our design does not allow us to confirm this. Future research might therefore specifically target reward value ratings in autism groups to shed more light on this effect. Also, our design included a task ensuring a sense of agency, so that the feedback took on a role of a reinforcer rather than introduced entirely new information. It would be interesting to systematically study the effect of expected and unexpected feedback on SPN and feedback-related ERPs and that in future studies.

## 5 Conclusion

The present study provides evidence that autistic traits and autism are linked to preserved, albeit less neuronally efficient, reward processing of non-social and relevant social rewards. By using a relevant social context, we increased the reward value in the social condition and potentially mitigated otherwise atypical responsiveness to social rewards in autism and higher autistic traits. In this design, we observed that while there were no behavioural differences between autistic and non-autistic participants, autism was linked to enhanced neuronal and autonomic responses to both social and non-social rewards. In the light of behavioural similarities between the groups, and a linear relationship between behavioural manifestations of social difficulties and neuronal reward hyperresponsiveness, we conclude that processing of non-social and relevant social rewards in autism is preserved, although less neuronally efficient. With this, we offer a coherent and simple interpretation of the contradictory evidence from clinical practice and empirical research, suggesting that relevance of social rewards is the crucial, although neglected, element of reward processing in autism.

## Author contributions

All authors substantially contributed to the conception of the design, interpretation of the data, and editing the manuscript. M.M. collected the data, performed data pre-processing and analyses, and wrote the original draft of the manuscript. All authors approved the submitted version and agreed to be personally accountable for their own contributions.

## Data availability statement

Data, analysis code, and an html file including all analyses’ steps and results can be found at https://osf.io/vse38/. The methods, hypotheses, and analyses were preregistered at https://osf.io/3re72.

## Funding

This work was supported by funding from the Berlin School of Mind and Brain, Humboldt-Universitaet zu Berlin and the German Academic Exchange Service (Deutsche Akademische Austauschdienst; DAAD) awarded to M.M.

## Declaration of Competing Interest

The authors declare that they have no known competing financial interests or personal relationships that could have appeared to influence the work reported in this paper.

## Data Availability

The data are available at the OSF repository (details in the manuscript).
