# Supplementary material for: Reward responsiveness in autism and autistic traits – Evidence from neuronal, autonomic, and behavioural levels
Source: Neuroimage Clin. 2023 May 24;38:103442. doi: 10.1016/j.nicl.2023.103442 (PMC10250120; doi:10.1016/j.nicl.2023.103442)
Supplement: Supplementary data 1 [file mmc1.pdf]

Supplementary material to

Reward responsiveness across autism and autistic traits. Evidence from neuronal, autonomic, and behavioural levels

Magdalena Matyjek, Mareike Bayer, and Isabel Dziobek

Supplementary Tables

Table S1. Means and standard deviations for artifact-free ERP and pupil responses trials, across conditions and phases.

	ERPs	Pupil responses
**Early anticipation**		
N	94 (8.11)	87 (15.45)
M	95 (5.83)	87 (13.13)
S	95 (6.08)	87 (13.19)
**Late anticipation** (successful trials)		
N	55.66 (6.37)	55 (9.77)
M	58.66 (4.86)	53 (8.27)
S	57.19 (5.34)	53 (7.96)
**Reception** (successful trials)		
N	55.46 (6.89)	55 (9.76)
M	58.61 (4.87)	53 (8.27)
S	57.18 (5.34)	53 (7.96)
**Statistical comparisons** (Testing whether the number of artifact-free segments between conditions for each phase varies statistically significantly)	No significant differences between conditions in either of the phases (all $F < 1.17$, $p > .31$	No significant differences between conditions in the early anticipation signals, $F(2,130) = 0.07$, $p = .936$. Differences in late anticipation, $F(2,130) = 3.13$, $p = .047$, and reception, $F(2,130) = 3.11$, $p = .048$, but no contrasts survived corrections for multiple comparisons (all $p_{corr} >= .075$).

Table S2. Means and standard deviations for ERP amplitudes, pupil responses, and reaction times in groups, conditions, and phases.

Group	Condition	Early anticipation	Late anticipation	Reception
		**ERPs [μV]**		
		CNV	*SPN*	*P3*
	N	-0.32 (1.53)	-4.4 (3.42)	2.98 (1.98)
ASC	*M*	-0.61 (1.77)	-4.34 (3.05)	3.33 (1.89)
	S	-0.49 (1.42)	-4.78 (3.1)	4.23 (1.94)
	N	-0.21 (1.39)	-2.95 (3.34)	1.73 (1.74)
HAQ	*M*	-0.16 (1.71)	-2.97 (3.07)	2.01 (1.83)
	S	-0.67 (1.82)	-3.52 (3.47)	2.81 (1.93)
	N	0.33 (1.23)	-3.3 (2.81)	2.23 (1.99)
LAQ	*M*	-0.1 (1.17)	-3.53 (2.45)	3.15 (2.16)
	S	-0.41 (1.13)	-3.94 (2.71)	3.82 (2.29)
		**Pupil sizes [au]**		
	N	0.11 (0.15)	0.24 (0.26)	-0.49 (0.65)
ASC	*M*	0.11 (0.14)	0.23 (0.25)	-0.38 (0.55)
	S	0.17 (0.19)	0.3 (0.25)	-0.4 (0.65)
	N	0.05 (0.29)	0.2 (0.23)	-0.19 (0.48)
HAQ	*M*	0.06 (0.26)	0.22 (0.31)	-0.14 (0.5)
	S	0.07 (0.38)	0.24 (0.36)	-0.15 (0.59)
	N	0.08 (0.21)	0.23 (0.29)	-0.1 (0.56)
LAQ	*M*	0.06 (0.17)	0.28 (0.27)	-0.07 (0.6)
	S	0.1 (0.29)	0.27 (0.25)	-0.17 (0.7)
		**Reaction times [ms]**		
	N		291.59 (49.65)	
ASC	*M*		292.79 (48.36)	
	S		296.14 (44.97)	
	N		290.34 (50.79)	
HAQ	*M*		270.16 (35.95)	
	S		289.4 (62.21)	
	N		292.52 (41.73)	
LAQ	*M*		283.48 (40.1)	
	S		290.88 (39.83)	

Debriefing questions

Participants answered the following debriefing questions: *How motivated were you in the experiment?* (general motivation); *How important was the reward type to you?* (importance of condition); *How often, right after giving the response, did you feel you knew whether you were successful?* (sense of agency), *How motivating did you find the cues?* (motivational value of cues); *How rewarding did you find the feedback pictures?* (rewarding value of feedback).

We found no significant differences in self-reported general motivation during the experiment across autistic traits, $r(79) = .05$, $p = .69$, or between groups (both $t <= 0.63$, $p >= .535$). In contrast, the type of reward was reported to be more important for those with less autistic traits, $r(79) = -.37$, $p < .001$, and for LAQ than ASC ($t = 3.5$, $p < .001$; in LAQ vs. HAQ, $t = -0.64$).

Three participants reported that they never or almost never knew whether they were successful in the game directly after giving response, while the rest reported they knew sometimes (16), often (24), most of the time (33), or always (3). This did not differ significantly between the groups (both $p >= .09$).

Figure S1 displays average ratings of motivational values of the cues and of reward values of the feedback stimuli across groups. There was a statistically significant interaction of group and condition on subjective ratings of cues' motivational values, $F(4,152) = 2.77$, $p = .03$. Although all groups showed descriptively higher ratings for the rewarded conditions (S and M) than N, post-hoc tests revealed that this was statistically significant in the LAQ and HAQ groups (all *est* >= 22.89, $p_{corr} < .001$) but not in the ASC group (all *est* >= 10.73). Moreover, LAQ on average rated S cues higher than ASC (*est* >= 23.96, $p_{corr} = .015$).

For the analysis of subjective reward value of the feedback stimuli, we built a linear mixed model with group, condition, and outcome (successful or unsuccessful trial) as main predictors and with their interactions. This model yielded significant interactions of group and outcome, $F(2,380) = 11.1$, $p < .001$, and of condition and outcome, $F(2,380) = 11.33$, $p < .001$. Post-hoc tests revealed that the ratings of positive outcomes were higher in the non-autistic groups than in ASC (both *est* >= 11.81, $p_{corr} <= .008$) and, as expected, in all groups positive outcomes were rated higher than negative ones (all *est* >= 46.64, $p_{corr} < .001$). Feedback stimuli in all conditions were also rated higher in successful than unsuccessful trials (all *est* >= 44.39, $p_{corr} < .001$), and social and monetary rewards were rated higher than neutral positive outcomes (both *est* >= 18.89, $p_{corr} < .001$). For details and plots, see sections 4.6., 5.1.3., and 6.1.3. in the html file.

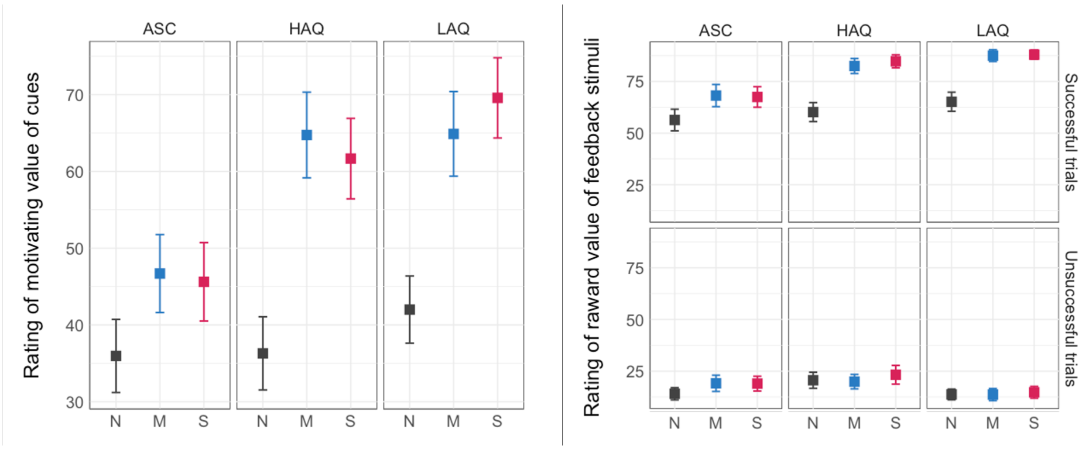

Figure S1. Average group ratings of (left) motivational value of the cues and (right) reward value of the feedback stimuli, separately for successful and unsuccessful outcomes.

Exploratory analyses

All steps of the following analyses can be found in the html file available in the corresponding repository (https://osf.io/vse38/).

4.9.Questionnaires and brain-behaviour correlations

Correlations between the questionnaires, ERPs, and pupil responses were calculated using Pearson's rank correlation coefficients.

1.1.1 Questionnaires

We explored correlations between the AQ and other questionnaires and found statistically significant correlations for LSAS-SR, $r(79) = .67$, $p < .001$, BIS scale, $r(79) = .45$, $p < .001$, BAS drive scale, $r(79) = -.23$, $p = .041$, BAS fun seeking scale, $r(79) = -.49$, $p < .001$, and BAS reward responsiveness, $r(79) = -.31$, $p = .006$. For details and plots, see section 4.5. in the html file. Altogether, these results suggest that higher autistic traits are related to decreased social ability, increased social anxiety, higher sensitivity of the inhibition system, and reduced activation of the approach system. Thus, these data support that individuals with high autistic traits have stronger behavioural motivation to move away from unpleasant stimuli than to move towards desired outcomes.

1.1.2 Correlations of the brain and pupil responses

Across all successful trials, the mean SPN correlated positively with the mean CNV, $r(79)= .27$, $p = .018$, and negatively with the P3, $r(79) = -.58$, $p < .001$. This suggests that larger brain responses in late anticipation are linked to also larger responses in early anticipation and in reception.

The mean pupil sizes were negatively correlated in reception and in early anticipation, *r*(66) = -.54, *p* < .001. This suggests that larger pupil responses in anticipation (i.e., more dilation) are related to larger pupil responses in reception (i.e., more constriction).

We found no significant correlations between ERPs and pupil sizes. Figure S2 shows correlogram of brain and pupillary responses. For details, see section 7.4. in the html file.

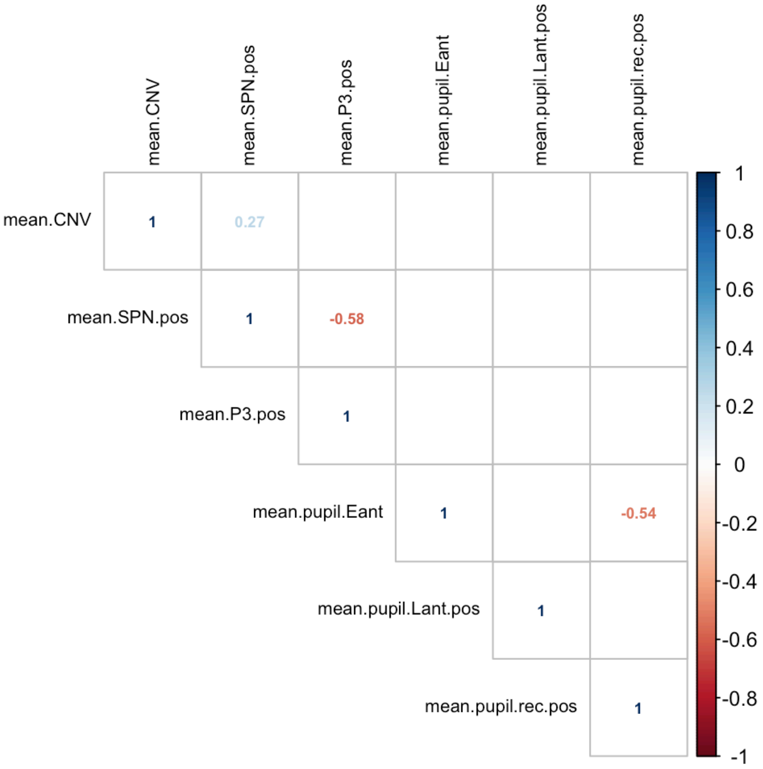

Figure S2. Correlogram: ERPs and pupil sizes in successful trials across conditions. Coefficients are displayed only for statistically significant correlations.

1.1.3 Correlations of brain, pupil, and self-reported data

The mean anticipatory brain responses (but not the P3) across conditions were found to correlate with the debriefing questions and questionnaires: Higher self-reported general motivation in the experiment was linked to larger CNV amplitudes, *r*(79) = -0.26, *p* = .019, higher BIS scores were linked to larger SPN amplitudes (in successful and unsuccessful trials, both *r*(79) >= -0.37, *p* <= .005), and higher BAS fun seeking scores in unsuccessful trials were linked to smaller SPN amplitudes, *r*(79) = 0.27, *p* = .017.

The mean pupil size across conditions correlated with self-reported importance of condition, so that the more important the condition, the weaker the pupil response in early anticipation and in reception of unsuccessful feedback (smaller pupil size in early anticipation, i.e., weaker dilations, and larger pupil size in reception, i.e., weaker

constrictions), respectively $r(66) = -0.25$, $p = .04$ and $r(66) = 0.34$, $p = .005$. For details, see section 7.3. in the html file.

4.10. Effects of group, condition, and outcome (successful and unsuccessful trials) on ERP and pupillary responses in the reception phase

To explore differences between reception of successful and unsuccessful outcomes on the neuronal and pupillary responses, we built models including group, condition, outcome (successful and unsuccessful), and their interactions. The predicted P3 and pupillary responses in successful and unsuccessful trials across all groups are shown in Figure S3 and Figure S4. Full analyses can be found in section 7.2. of the html file.

For neuronal responses, we observed an interaction of group and outcome ($F(2,395) = 3.53$, $p = .03$, $f_p = 0.13$). This was driven by larger P3 amplitudes in ASC as compared to HAQ, for both successful and unsuccessful trials (both $p_{corr} <= .034$). The pupillary data revealed significantly larger pupil constrictions to outcomes in successful than in unsuccessful trials, $F(1,513) = 31.24$, $p_{corr} < .001$, $f_p = 0.25$ (no other contrasts were significant).

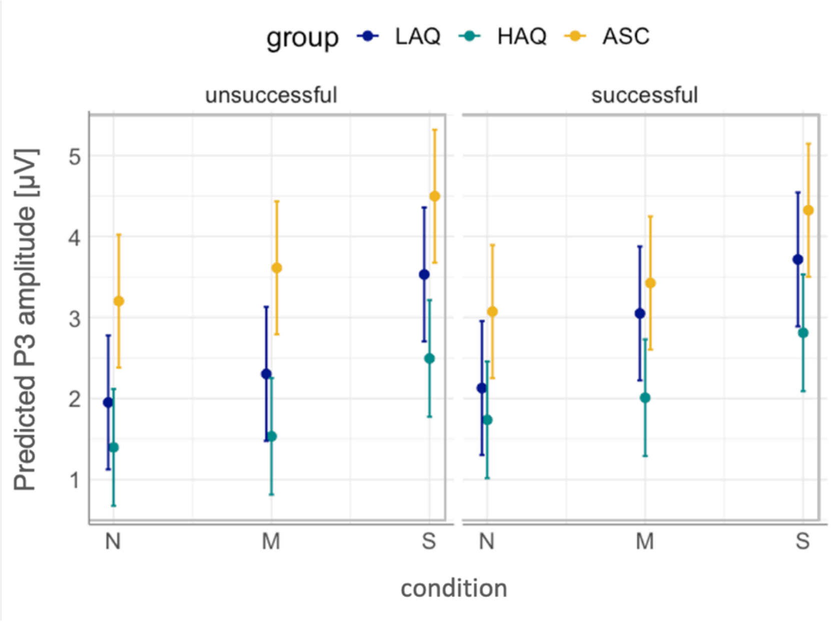

Figure S3. Average P3 responses in successful and unsuccessful trials across groups.

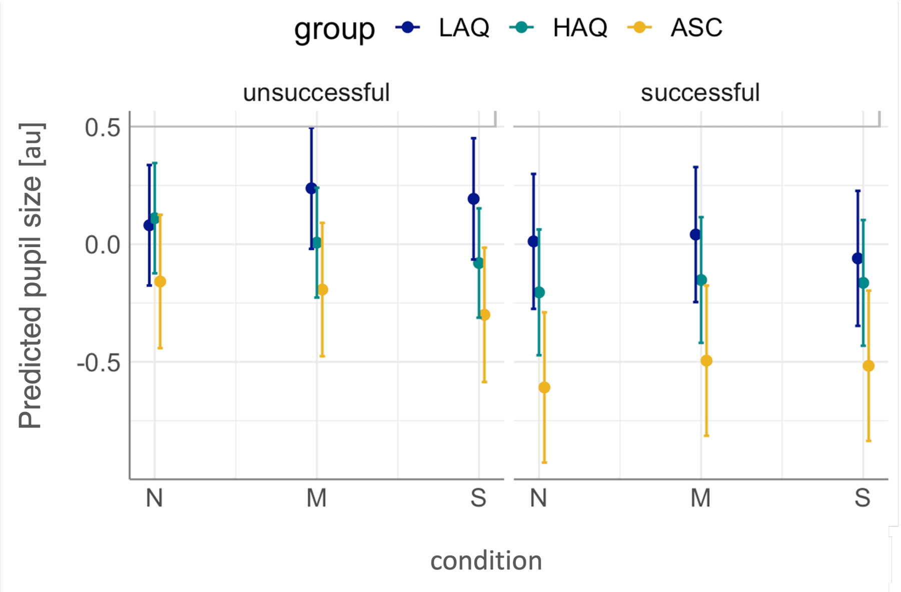

Figure S4. Average pupil sizes in successful and unsuccessful trials across groups.

4.11. Dimensional analyses – AQ as a predictor of reaction times, ERPs, and pupillary responses

In addition to the pre-registered analyses, we explored the effects of autistic traits across the sample on reward responses (a dimension analysis). Because in the spectrum view of autism the distribution of autistic traits in the general population is continuous, we conducted exploratory analyses in which the main predictor of reward responsiveness is continuous AQ instead of group. Finally, it is conceptually interesting to consider whether the potential atypicalities in reward processing would increase linearly with higher autistic traits in the population, or whether this increase would become steeper with particularly high trait levels (for example, around the approximate cut-off of autism diagnosis). To investigate this, we also built exploratory generalised additive mixed models (GAMMs) with package mgcv ver. 1.8-31 (Wood, 2011) in the following form:

DV ~ condition + s(AQ, by = condition) + LSAS-SR + s(subject, bs='re'),

where DV is the dependent variable, s(AQ, by = condition) is a smooth term for AQ fitted separately for each condition, and s(subject, bs='re') is the random smooth for subjects. AQ and LSAS-SR were centred before they entered the statistical models. Here, we report only the main effects of those additional models and the complete analyses can be found in the analysis code in the referred repository. Figure S5 shows predicted neuronal and pupillary responses across levels of autistic traits in all phases. All analysis steps are shown in section 7.1. of the html file.

1.1.4 ERPs

Condition was a significant predictor of the brain responses in all linear models with continuous AQ (instead of group): early anticipation, $F(2,158) = 7.76$, $p = .001$, $f_p = 0.31$, late anticipation, $F(2,156) = 3.57$, $p = .031$, $f_p = 0.21$, and reception, $F(2,158) = 4.32$, $p = .015$, $f_p = 0.23$. In all models, responses to S were larger than to N (all $p_{corr} <= .031$, *est* >= 0.46). Additionally, in late anticipation and reception, S elicited larger ERP amplitudes than M (both $p_{corr} <= .035$, *est* >= 0.47), and in reception M was linked to larger P3 than N ($p_{corr} =.002$, *est* = 0.51). The AQ score significantly predicted the brain responses only in the early anticipation, $F(1,79) = 4.28$, $p = .042$, $f_p = 0.23$ (in late anticipation and reception $f_p = 0.05$), with higher AQ scores linked to larger (more negative) CNV response.

GAMMs with continuous AQ score yielded similar pattern of effects: condition (entered as a parametrical term) significantly predicted ERP amplitudes in all models (all $F >= 4.26$, $p <= .012$) with larger responses to S than N. In the early anticipation model (and not late anticipation and reception), the AQ smooth was significant ($F = 4.12$, $p = .044$). Importantly, in all models AQ was fitted with effective degrees of freedom (edfs) of 1 (in reception edf = 1.2), which suggests that the best approximation of the relationship between autistic traits and reward-related brain responses is linear.

1.1.5 Pupil sizes

In both anticipation phases, the pupil sizes were predicted significantly by condition: early anticipation, $F(2,128) = 9.66$, $p < .001$, $f_p >= 0.39$, and late anticipation, $F(2,128) = 5.66$, $p = .004$, $f_p = 0.29$. Responses were larger in S than in N (both $p_{corr} <= .018$, *est* >= 0.04) and in early anticipation also in S than M ($p_{corr} < .001$, *est* >= 0.05). AQ approached significance in reception, $F(1,42) = 3.33$, $p = .075$, $f_p = 0.33$ (in anticipation phases both $f_p <= 0.29$).

GLMMs yielded a main effect of condition in both anticipatory phases (both $F >= 5.41$, $p <= .005$) with larger responses (more dilation) to S than N. In all phases, the models fit better without separate AQ smooths for conditions and with edf = 1. AQ was statistically significant only in reception with higher AQ scores linked to smaller pupil sizes ($F = 6.44$, $p = .012$).

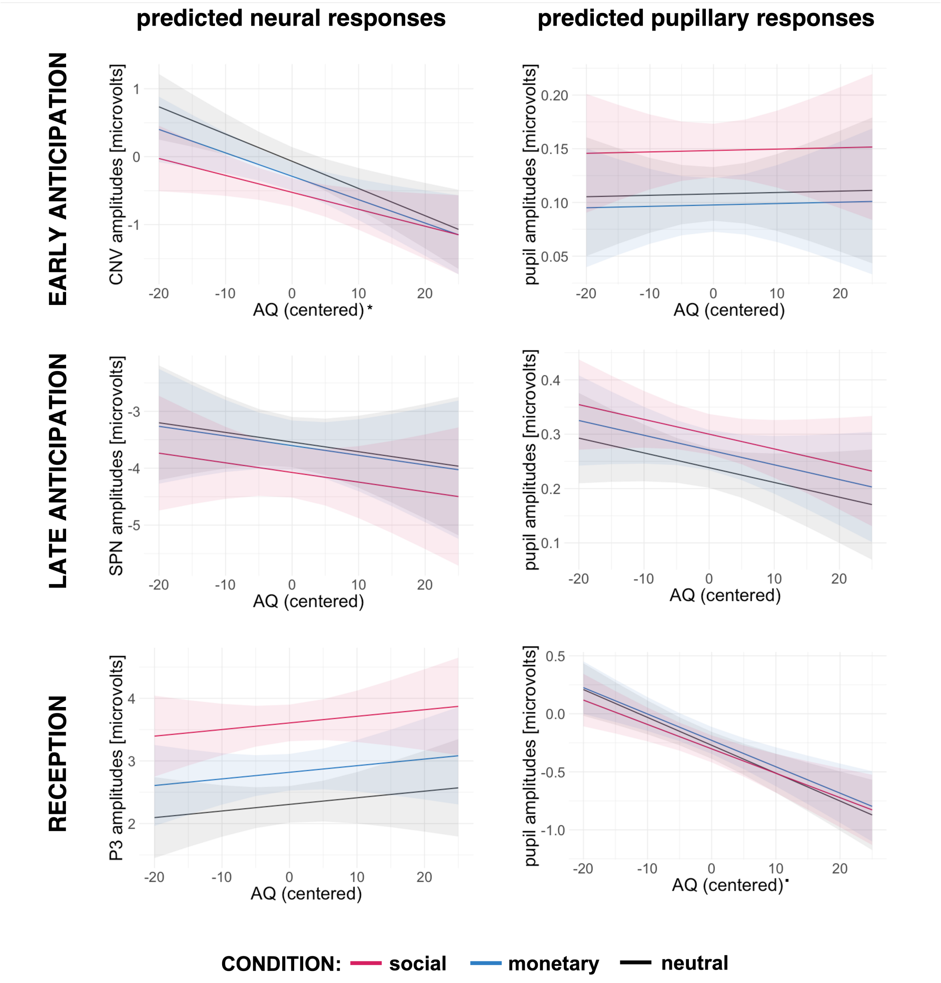

Figure S5. Predicted values of neuronal and pupillary responses to social, monetary, and neutral outcomes in successful trials across autistic traits (AQ) in all participants. Shadowed areas represent 95% confidence intervals. Statistically significant effects of AQ were marked with * for p < .05.

1.1.6 Reaction times corrected for accuracy

Condition was a significant predictor of the LISAS scores, $F(2,136) = 8.6$, $p < .001$, $f_p = 0.36$, with faster responses to M than to S and N (both $p_{corr} <= .001$, $est >= 6.31$). The AQ did not significantly predict LISAS ($f_p = 0.04$). Figure S6. shows predicted LISAS scores across AQ.

The GAMM model showed a slightly better fit for a separate smooth for AQ in each condition, with edf = 1 for N and M and 1.3 for S. However, none of the smooths were significant. Condition as a parametric term significantly predicted LISAS ($F = 8.62$, $p < .001$), with the fastest responses in M.

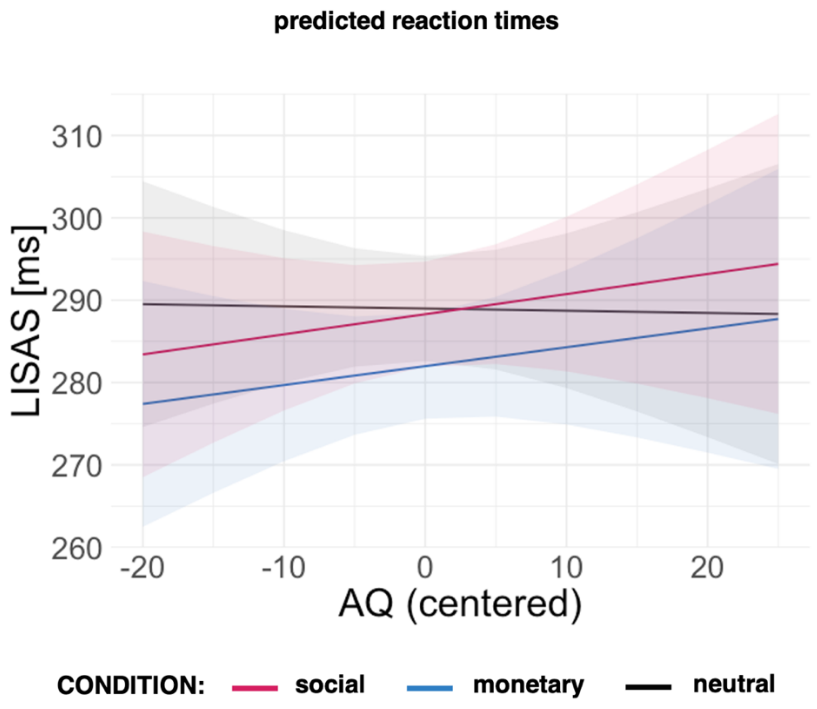

Figure S6. Predicted reaction times corrected for accuracy in social, monetary, and neutral conditions in successful trials across autistic traits (AQ) in all participants. Shadowed areas represent 95% confidence intervals.

4.12. Age and gender effects in ERP and pupillary models

For all primary models, we additionally explored the effects of age and gender. New models were in the form:

DV ~ group * age + group * gender + condition + LSAS-SR + (1 | subject).

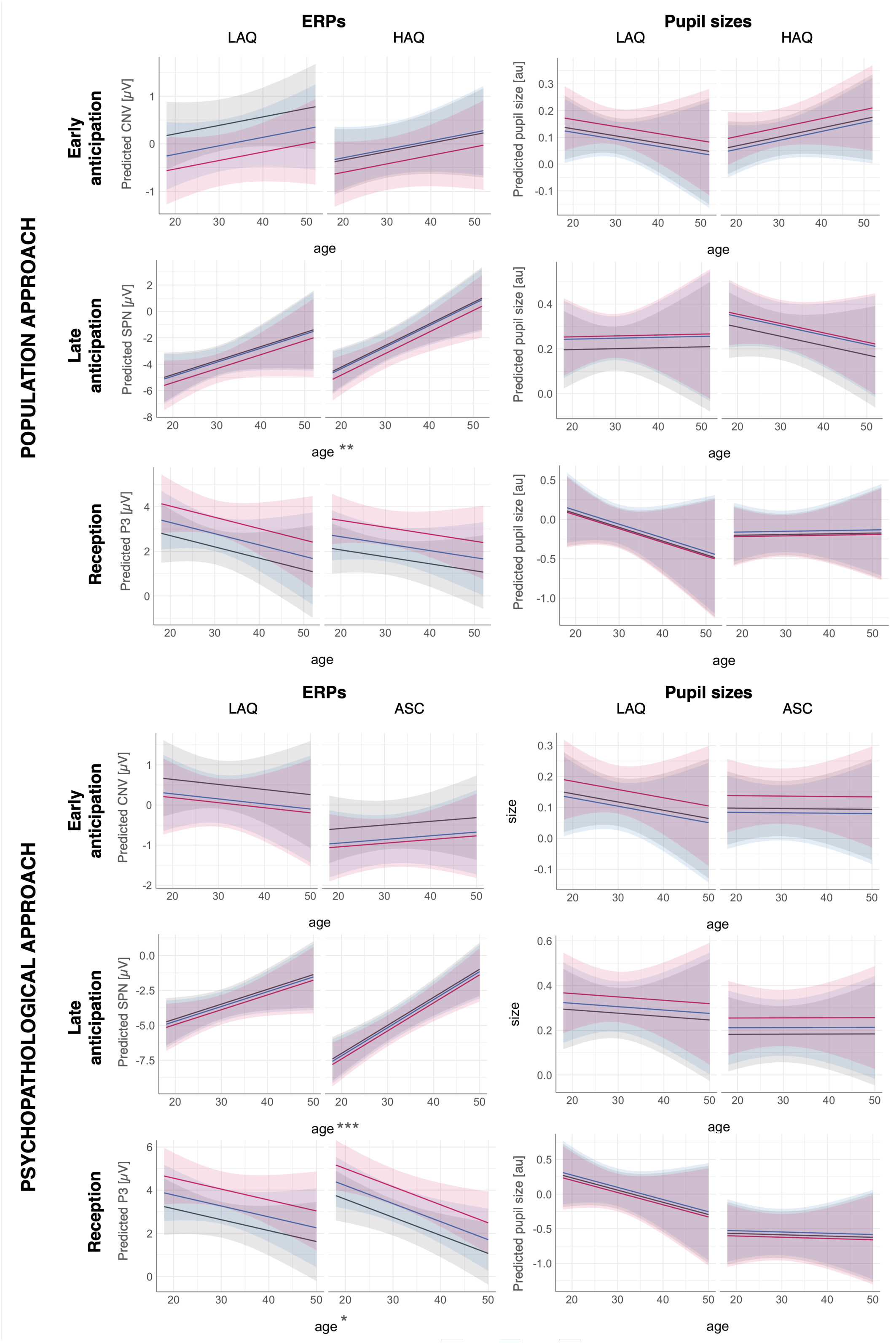

Figure S7. Effects of age on ERP and pupillary responses across groups (LAQ, HAQ, ASC), conditions (N = neutral, M = monetary, S = social), and phases (early / late anticipation, reception). Statistically significant predictors are marked with * for p<.05, ** for p<.01, and *** for p<.001. Details and full analyses can be found in the html file in sections 5.2-5.3 and 6.2-6.3.

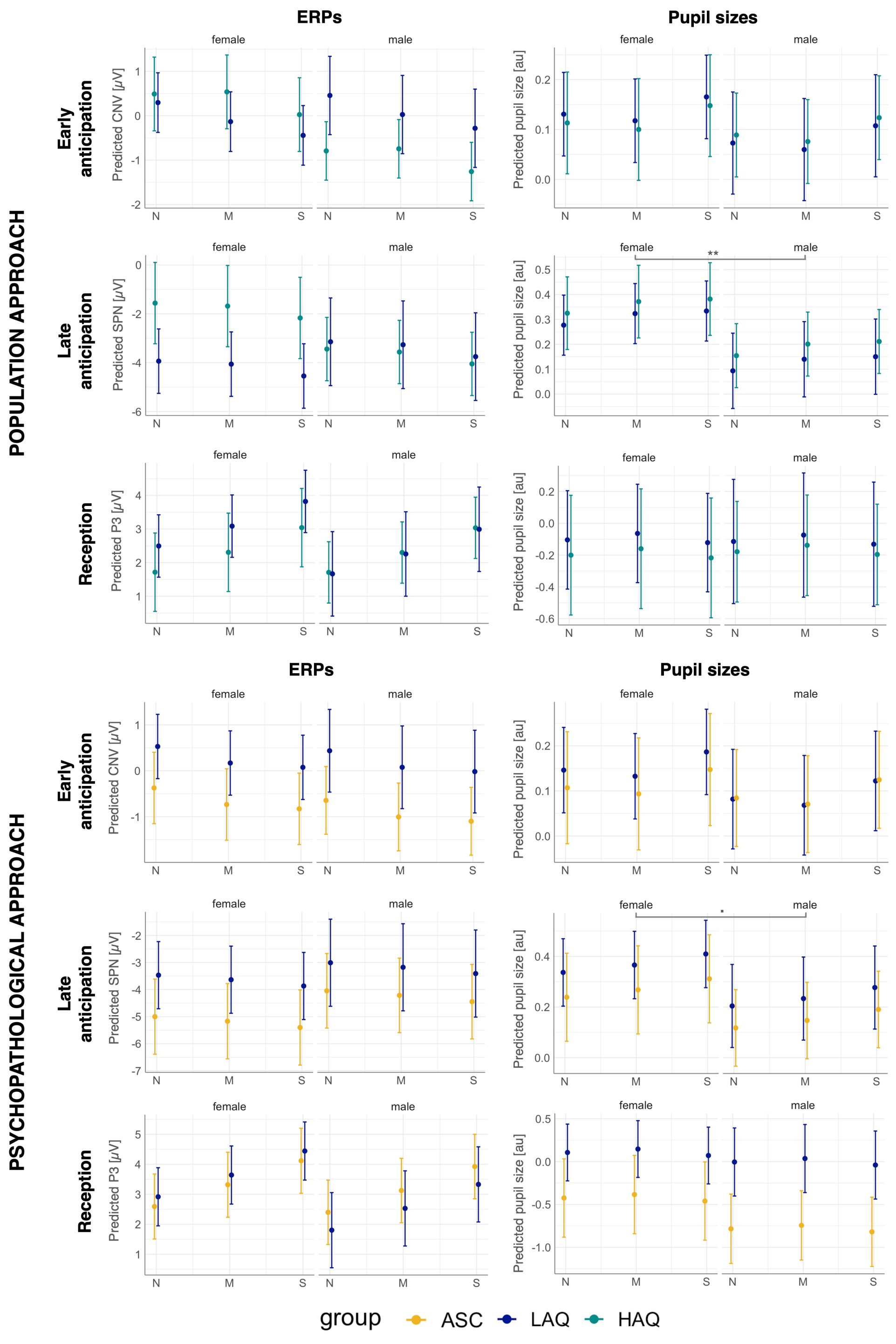

Figure S8. Effects of gender on ERP and pupillary responses across groups (LAQ, HAQ, ASC), conditions (N = neutral, M = monetary, S = social), and phases (early/late anticipation, reception). Statistically significant predictors are marked with * for p<.05, ** for p<.01, and *** for p<.001. Details and full analyses can be found in the html file in sections 5.2-5.3 and 6.2-6.3.
